# Laboratory Synthesis, Characterization, and Py-GCMS-Based ^13^C Quantification of ^13^C‑Enriched Polyethylene

**DOI:** 10.1021/acsomega.6c00354

**Published:** 2026-05-05

**Authors:** Ravindra Reddy Chowreddy, Alireza Hassani, Gustav Vaaje-Kolstad, Bavan Mylvaganam

**Affiliations:** † Norner Research AS, Dokkvegen 20, 3920 Porsgrunn, Norway; ‡ Faculty of Chemistry, Biotechnology and Food Science, Norwegian University of Life Sciences, 1433 Ås, Norway

## Abstract

Labeling polymers with ^13^C isotopes enables precise tracking of carbon during chemical transformations and biodegradation, offering valuable insights into degradation mechanisms and the environmental impact. Nevertheless, the commercial availability of such labeled polymers remains limited. This is primarily due to the lack of ^13^C-labeled monomers required for synthesizing the corresponding polymers and the high costs associated with their production. These challenges hinder the widespread application of ^13^C-labeled polymers in research and industry. In this study, we report the successful laboratory synthesis and characterization of ^13^C stable isotope-enriched polyethylene. Homopolyethylene and copolyethylene samples with an approximate 25 wt % ^13^C-enrichment were produced by polymerizing mixtures of standard and ^13^C isotope-labeled ethylene monomers. The characterization of the ^13^C-labeled polyethylene samples indicated that their microstructural properties were similar to those of the standard polyethylene samples produced from conventional ethylene monomers. Additionally, we present the quantification of ^13^C isotope enrichment in polyethylene using pyrolysis gas chromatography–mass spectrometry (Py-GCMS), alongside conventional nuclear magnetic resonance (NMR) and isotope ratio mass spectrometry (IRMS) analysis. Our results demonstrate that the ^13^C isotope enrichment values measured by Py-GCMS are comparable to those obtained from the conventional IRMS method, indicating the potential application of Py-GCMS as an alternative method for ^13^C isotope analysis in polymer samples.

## Graphical abstract

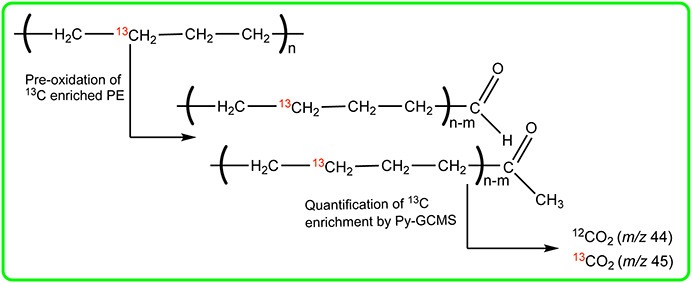

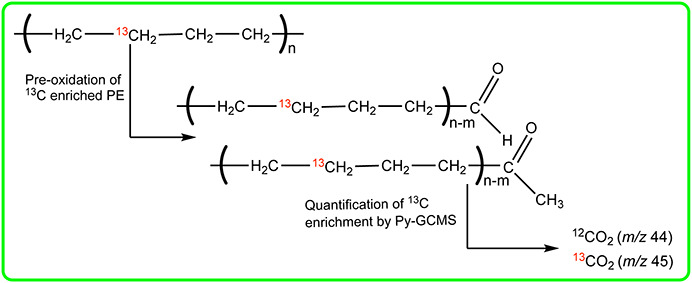

## 1 Introduction

The increasing concern about the leaching of micro- and nanoplastics into the environment has raised fundamental questions about their potential impact on human health, wildlife, and entire ecosystems.^1–7^ There is growing concern among both legislative bodies and consumers about identifying the sources of micro- and nanoplastics, assessing their potential health and environmental risks, and mitigating leakage throughout the value chain.^8–11^ A key to identifying microplastics in different environments is undoubtedly the use of advanced characterization methods. The rather low concentration and small particle sizes of micro- and nanoplastics (typically below 5 mm and between 1 mm and 2 nm, respectively) pose challenges for their identification and differentiation, especially in organic-rich media such as soil.

Pyrolysis gas chromatography–mass spectrometry (Py-GCMS) has been extensively used as a destructive analysis method in the research field of microplastic detection.^12–18^ This method enables the identification and quantification of microplastics in complex organic matrices by thermal decomposition of the polymer backbone into characteristic fragments for further precise mass spectroscopic analysis. Despite the great advantage in sample preparation, the complex nature of the matrices containing microplastics can introduce complications. In addition, distinguishing degradation products of plastics from biogenic substances naturally present in the studied matrix can be challenging when interpreting the obtained mass spectra. As a result, labeling polymers with carbon isotopes for tracking atoms and molecules originating from plastic materials offers distinct advantages.

Carbon is particularly useful for isotopic tracing, as it exists in ^14^C radioactive isotopic and ^13^C stable isotopic forms. Labeling polymers with one of these carbon isotopes allows selective tracking of carbon during chemical transformations and biodegradation into low molar mass compounds. This method of isotopic labeling for investigating polymer biodegradability has been known since the early 1970s. Initial studies focused on ^14^C radioisotope labeling and tracer analysis for evaluation of biodegradation of synthetic polymers.^19–21^ However, methodologies involving radioisotope-labeled materials have certain drawbacks, including high costs, stringent safety and handling regulations, and the need for specialized instrumentation dedicated to analyzing radioactive ^14^C-containing samples.^22,23^

Labeling of polymers with the ^13^C isotope for tracking carbon during chemical transformations and biodegradation offers certain advantages. The ^13^C isotope is stable, does not lead to radioactive contamination, and is not subject to restrictive regulations.^22^ In addition, ^13^C-labeled monomers are relatively inexpensive and readily available for the synthesis of polymers. Consequently, several researchers have utilized ^13^C stable isotope labeling in various polymer systems to assess chemical transformations and biodegradation.

Recently, the biodegradation of ^13^C-labeled biodegradable plastics, such as poly­(butylene succinate)^24^ and poly­(butylene adipate-*co*-terephthalate),^25^ in soil has been investigated by tracking carbon dioxide (CO_2_), microbial biomass, and mineralization. Additionally, several conventional polymers, such as polyethylene,^26,27^ polystyrene,^28^ and polyacrylate,^29^ labeled with ^13^C isotopes, have also been investigated for biodegradation. Furthermore, the chemical transformation mechanisms of polypropylene^30–33^ and nylon-6,6 ^34,35^ during various physical degradation processes, such as oxidation, thermal degradation, and γ radiation, have also been investigated by tracing the labeled products.

Polyethylene (PE), as the most dominant polymer among all synthetic polymers, serves as an ideal model for understanding structural composition and degradation mechanisms due to its simple structure.^36^ In this study, we report the synthesis of ^13^C isotope-enriched PE and the characterization of its structural and morphological properties compared to their naturally abundant counterparts. Further, Py-GCMS was explored as an analytical tool to determine ^13^C/^12^C ratios in ^13^C-enriched polyethylene materials. Py-GCMS generates a pyrolytic fingerprint, revealing patterns like triplet distributions of diolefins, olefins, and *n*-paraffins, which are markers of PE fragmentation. These fingerprints are useful for polymer type identification and degradation assessment. However, determining ^13^C/^12^C isotope ratios in neat polymers with Py-GCMS is challenging due to complex fragment mixtures and low molecular ion intensity.^37,38^ To address this, a mild oxidation step was introduced before pyrolysis. This step mimics environmental weathering and produces more complex degradation products, aiding in the assessment of specific ion distributions and degradation mechanisms. The pyrolysis step also generates CO_2_ from the polymer, which is crucial for determining ^13^C/^12^C isotope ratios. This dual-purpose approach combines structural degradation profiling with isotope quantification, thereby enhancing the applications of Py-GCMS for material characterization and isotope-labeled polymer research. Validation was performed using isotope ratio mass spectrometry (IRMS) to benchmark the results.

## 2 Materials and Methods

### 2.1 Materials

Industrial-grade standard ethylene monomer and nitrogen (Grade 6) were kindly provided by INEOS Bamble AS, Norway. These standard ethylene and nitrogen gases were purified to polymerization grade by passing them through molecular sieve columns. Ethylene was passed through 13X molecular sieves, while nitrogen was purified using 3 Å and 4 Å molecular sieve columns. The molecular sieves were purchased from Sigma-Aldrich. Polymerization-grade *n*-hexane was provided by SCG Chemicals, Thailand, and 1-hexene was provided by Borealis AG, Austria, both of which were free samples and used without further purification. Hydrogen gas (Grade 6) was supplied by Nippon Gases, Norway. The cocatalyst, triethylaluminum (TEAL), and the ^13^C-labeled ethylene monomer, ethylene-^13^C_1_ (H_2_C = ^13^CH_2_) with 99% ^13^C purity, were purchased from Sigma-Aldrich. The Zeigler–Natta (ZN) catalyst was synthesized in the laboratory according to the literature.^39^

### 2.2 Methods

#### 2.2.1 Preparation of Polyethylene Samples

Four different types of PE materials were synthesized by ZN-catalyzed polymerization in a high-pressure reactor (see below for details). Of these, two were homopolyethylene materials, one conventional reference (sample name: ^12^C–H-PE) and another with ^13^C-enrichment (sample name: ^13^C–H-PE), and the other two were copolyethylene materials with a hexene comonomer, one conventional reference (sample name: ^12^C–C-PE) and another with ^13^C-enrichment (sample name: ^13^C–C-PE) (Table 1). The plausible polymer structures for visualization purposes are listed in Figure 1.

**Table 1:** Target Polyethylene Types and Monomer Compositions

run #	target polyethylene	ethylene monomers		comonomer	comment
		standard ethylene, H_2_C = CH_2_ (vol %)	ethylene-^13^C_1,_ H_2_C = ^13^CH_2_ (vol %)		
1	^12^C–H-PE	100	0	none	reference homopolyethylene
2	^13^C–H-PE	50	50	none	^13^C-enriched homopolyethylene
3	^12^C–C-PE	100	0	1-hexene	reference copolyethylene
4	^13^C–C-PE	60	40	1-hexene	^13^C-enriched copolyethylene

**Figure 1:**
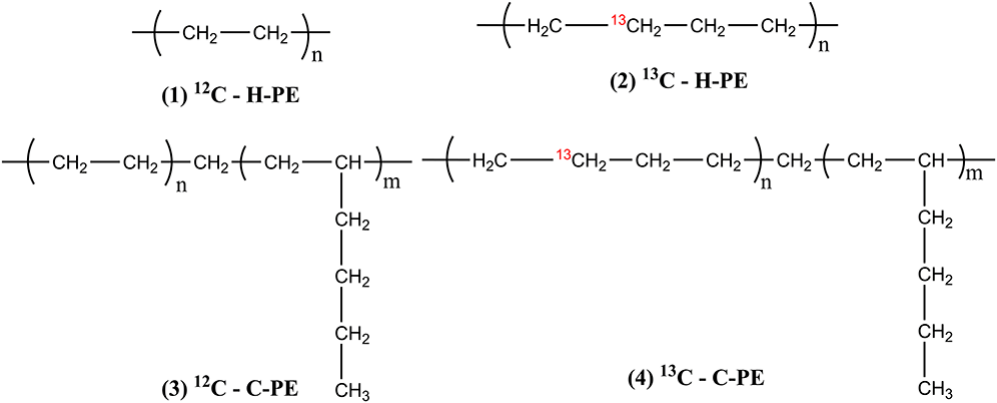
Plausible chemical structures of high-density polyethylene ((1) and (2)) and linear low-density polyethylene ((3) and (4)) samples produced from standard and ^13^C-enriched monomers. The chemical structures presented are strictly not stoichiometric.

The polymerizations were carried out in a 3000 mL high-pressure stainless-steel reactor with automatic temperature control and mechanical stirring. Before the polymerization runs, the reactor was vacuum-dried for 1 h at 150 °C and then cooled to room temperature. First, 500 mL of *n*-hexane was transferred to the reactor. Later, 8 mg of the ZN catalyst and 0.2 mL of the TEAL cocatalyst, premixed in 2 mL of *n*-hexane, were transferred into the reactor, followed by heating of the reactor to 80 °C. Handling of both the catalyst and cocatalyst was performed in a glove box under a nitrogen atmosphere. To produce the reference homopolyethylene sample, ^12^C–H-PE, from the conventional monomer, a mixture of ethylene at 12.6 bar and hydrogen at 6 bar was prepared at room temperature and introduced into the reactor at a partial pressure of 1.7 bar at 80 °C. Polymerization was then carried out at 80 °C for 1 h with stirring at 450 rpm. The total pressure in the reactor was maintained at 3.0 bar. At the end of the reaction, the reactor was vacuum-dried to remove *n*-hexane, and the white polymer powder was recovered.

In the polymerization experiment intended to produce ^13^C–H-PE, a ^13^C-enriched PE sample, all of the polymerization conditions were kept similar to those for ^12^C–H-PE except for the monomer feed. To produce the ^13^C-enriched PE sample, a mixture of standard ethylene and ethylene-^13^C_1_ was first premixed at a 50:50 volume ratio. Later, a mixture of the ethylene monomers and hydrogen was prepared, similar to what was done to produce ^12^C–H-PE, and polymerization was conducted. In a couple of copolymerization trials, one with standard ethylene alone (^12^C–C-PE) and another with a mixture of standard ethylene and ethylene-^13^C_1_ (^13^C–C-PE), the comonomer 1-hexene (2 mL corresponding to its density at room temperature) with ^13^C natural abundance was also introduced into the reactor to produce an ethylene–hexene copolymer. The monomer feed composition to produce ^12^C–C-PE and ^13^C–C-PE was similar to that of ^12^C–H-PE and ^13^C–H-PE, respectively, except for the additional comonomer hexene and the 60:40 ratio of conventional ethylene to ethylene-^13^C_1_ for the preparation of ^13^C–C-PE. All other polymerization conditions were kept the same.

#### 2.2.2 Size Exclusion Chromatography (SEC)

The weight-average molar mass (Mw), number-average molar mass (Mn), and the molar mass distribution in the PE materials were determined using a GPC-IR5 system (Polymer Char, Valencia, Spain) equipped with four PLgel 20 μm MIXED-A columns (Agilent Technologies, Santa Clara, CA, USA). Approximately 4 mg of the PE powder was predissolved in 8 mL of 1,2,4-trichlorobenzene at 160 °C for 3 h, and 200 μL samples were injected into the SEC system. The analysis was performed at 150 °C using a high-sensitivity infrared detector and 1,2,4-trichlorobenzene as a mobile phase (1 mL/min flow rate). Polystyrene standards with a narrow molecular mass distribution and Mpeak in the range of 1140–7,500,000 g/mol (Agilent Technologies, Santa Clara, CA, USA) were used for calibration.

#### 2.2.3 Differential Scanning Calorimetry (DSC) Analysis

The crystallinity of polymer resins was assessed by performing DSC analysis using a TA DSC250 instrument. A compression-molded film sample with a thickness of 0.2 mm was utilized for analysis. About 3–5 mg of the sample was encapsulated into sealed aluminum pans and analyzed. The DSC analysis involved three cycles of heating and cooling: (1) heating the sample from −10 to 200 °C at a heating rate of 10 °C/min and holding for 5 min, (2) cooling from 200 to −10 °C at a cooling rate of 10 °C/min and holding for 5 min, and (3) heating the sample from −10 to 200 °C at a heating rate of 10 °C/min. The melt temperature (*T*_m_), crystallization temperature (*T*_c_), and enthalpies of melting (Δ*H*_m_) were evaluated from the second heating segment of DSC analysis. The percentage of crystallinity (*X*_c_) in the PE resins was determined according to the following equation:

$$X_{c}(\%)=\frac{\Delta H_{m}}{\Delta {H_{f}}^{0}}\times 100$$

where Δ*H*_f_^0^ is a reference value for the enthalpy of melting of 100% crystalline material: 293 J g^–1^.^40^ For each sample, the analysis was carried out in duplicate, and average values have been reported.

#### 2.2.4 Thermogravimetric Analysis (TGA)

TGA of the PE powder samples was carried out using a Q5000IR instrument from TA Instruments. TGA analysis was performed to determine the thermal stability and amount of incombustible inorganic material in the PE samples. About 10 mg of the sample was subjected to TGA. TGA was performed on the samples from room temperature to 600 °C under a nitrogen atmosphere and from 600 to 1000 °C under an air atmosphere with a heating rate of 20 °C min^–1^.

#### 2.2.5 Fourier Transform Infrared Spectroscopy (FTIR)

FTIR analyses were performed on the PE samples using a PerkinElmer Spectrum TWO instrument equipped with a Spotlight 200i microscope. Specimens for FTIR analysis were prepared by hot-pressing powder into 0.2 mm thick film discs. Typically, 32 scans were used for spectral averaging at a resolution of 2 cm^–1^. FTIR spectra were recorded in the transmission mode between 4000 and 500 cm^–1^.

#### 2.2.6 ^13^C NMR Characterization

^13^C NMR analysis was conducted to determine the comonomer content in copolymer samples and to evaluate ^13^C-enrichment in the ^13^C-enriched PE samples. A Bruker Avance III 400 spectrometer, equipped with a 5 mm high-temperature cryoprobe and a robotic sample changer with a preheated carousel, was used for the analysis. Approximately 20–25 mg of polyethylene samples was dissolved in 0.5 mL of tetrachloroethane-1,2-d2 by heating at 120 °C. The solution was loaded into the carousel, which was maintained at the same temperature. The spectra were recorded under the following conditions: 45° pulse; 2.83 s acquisition time; 5.0 s relaxation delay; 250 scans.

#### 2.2.7 IRMS Analysis

IRMS is a specialized analytical method commonly used to measure stable isotope ratios in a wide variety of samples.^41,42^ The ^13^C/^12^C isotope ratio in the synthesized PE samples was determined using a Thermo Scientific Delta V Advantage IRMS. The IRMS was coupled to a Thermo Scientific Elemental Analyzer IsoLink through a Thermo Scientific ConFlo IV interface for continuous flow measurements. For analysis, samples ranging from 20 to 150 μg were combusted at 1020 °C in an elemental analyzer, using a pulse of O_2_ to produce CO_2_ gas for isotopic analysis. The resulting CO_2_ was transferred through the ConFlo interface to the mass spectrometer for the measurement of the isotopic composition. The *m*/*z* values expected were 44 for ^12^C^16^O^16^O, 45 for ^13^C^16^O^16^O and ^12^C^16^O^17^O, and 46 primarily for ^12^C^18^O^16^O. The isobaric interference of ^12^C^16^O^17^O for *m*/*z* = 45 was corrected using the “^17^O correction” algorithm implemented in the IRMS software, Qtegra from Thermo Scientific. The software applies calculations for “^17^O correction” as described by others.^43^ The ^13^C/^12^C ratio and ^13^C-enrichment in the PE materials were calculated from the δ^13^C recorded by the IRMS instrument relative to the international standard Vienna Pee Dee Belemnite (VPDB). The precision and accuracy of the instrument were evaluated using repeated measurements of reference standards with a reproducibility of ± 0.1‰ for δ^13^C. CO_2_ was used as the reference gas, and IAEA-600 served as the calibration standard.

#### 2.2.8 Pyrolysis GCMS

Pyrolysis GCMS (Py-GCMS) analysis was performed on ^13^C-labeled and nonlabeled PE materials to determine ^13^C/^12^C isotope ratios. For this analysis, 100–200 mg of each sample was placed in deactivated stainless-steel sample cups and introduced into a multishot EGA/PY-3030D Pyrolyzer (Frontier Laboratories Ltd., Fukushima Japan) coupled with a 7890N gas chromatogram and a 5975 mass spectrometer (Agilent Technologies, Santa Clara, CA, USA). Both untreated and thermally oxidized PE powders were used for Py-GCMS analysis. In the case of Py-GCMS analysis of thermally oxidized PE, ∼100 mg of the powder samples was added to the deactivated stainless-steel sample cups, and oxidation was performed in a hot-air oven at 200 °C for 70 min.

The experimental conditions for the pyrolysis were as follows. The preselected pyrolysis temperature was set at 600 °C. The interface between the pyrolysis furnace and the GC–MS system was set at 200 °C. The GC injector was operated in the split mode (100:1 ratio) at 300 °C. The analysis of pyrolysis products was performed on an Ultra-Alloy metal capillary column containing 5% diphenyl and 95% dimethylpolysiloxane stationary phase (30 m, 0.25 mm ID, 0.25 mm, Frontier Laboratories Ltd., Japan). The chromatographic conditions were 70 °C, 20 °C/min to 350 °C for 16 min carrier gas (He, 5.0) flow: 1.0 mL/min. MS parameters: electron impact ionization (EI, 70 eV) in the positive mode; ion source temperature 230 °C; scan range 29–350 *m*/*z*; interface temperature 300 °C. Agilent MassHunter and F-search (Frontier Laboratories Ltd.) software were used for data analysis, and peak assignments were based on mass spectra libraries (NIST MS Search 2.4 and F-search Ver. 3.7.1).

## 3 Results and Discussion

### 3.1 Polymer Preparation and Characterization

The purpose of this study was to produce polyethylene samples with and without ^13^C-enrichment with significantly different microstructures (Figure 1). These polyethylene variants were successfully synthesized and characterized. Table 2 presents the monomer compositions, catalyst productivity, and properties of the produced polyethylene samples. The polymerization runs conducted to produce both homopolyethylene and copolyethylene samples using the standard ethylene monomer (^12^C–H-PE) resulted in productivities of 310 and 500 g PE/g cat h, respectively. Both homopolyethylene and copolyethylene samples with ^13^C-enrichment were also synthesized by incorporating the ^13^C-labeled ethylene monomer (H_2_C = ^13^CH_2_) along with a standard ethylene monomer (^13^C–H-PE), and these runs resulted in productivities of 630 and 400 g PE/g cat h, respectively. These results indicate that the ZN catalyst is equally effective in polymerizing standard and ^13^C-labeled ethylene.

**Table 2:** Summary of Monomer Composition, Catalyst (ZN) Productivity, and Properties of the Synthesized Polyethylene Materials

monomer composition/catalyst productivity/polyethylene properties	^12^C–H-PE	^13^C–H-PE	^12^C–C-PE	^13^C–C-PE
ethylene monomer (vol %)	C_2_H_4_ (100)	C_2_H_4_ (50) + H_2_C = ^13^CH_2_ (50)	C_2_H_4_ (100)	C_2_H_4_ (60) + H_2_C = ^13^CH_2_ (40)
comonomer (type)	none	none	1-hexene	1-hexene
catalyst productivity (g PE/g cat h)	310	630	500	400
weight-average molar mass, *M*_w_ (g/mol)	130,950	80,200	145,800	117,350
number-average molar mass, *M*_n_ (g/mol)	15,000	10,700	23,050	20,200
dispersity, *Đ*	8.7	7.5	6.4	5.8
melting temperature, *T*_m_ (°C)	135	133	131	131
crystallization temperature, *T*_c_ (°C)	118.5	118	115.5	116
degree of crystallinity, χ_c_ (%)	74	74.5	63.5	64
onset of decomposition (°C)	435	415	415	435
wt. loss between 23 and 600 °C (%)	99.86	99.82	99.85	99.75
residue at 1000 °C (%)	0.13	0.15	0.15	0.20
comonomer content (mol %)			0.3	0.2

The *M*_w_, *M*_n_, and dispersity (*Đ*) of polyethylene samples determined by SEC are presented in Table 2. Molar mass distribution curves are presented in Figure S1. Among the PE samples produced, homopolyethylene samples (^12^C–H-PE and ^13^C–H-PE) exhibited slightly lower molar masses (*M*_w_ and *M*_n_) than the copolymer samples (^12^C–C-PE and ^13^C–C-PE). The homopolyethylene produced with the standard ethylene monomer alone showed Mw and Mn values higher than those of polyethylene produced in the presence of ^13^C-containing ethylene. Similarly, among copolymer samples, ^12^C–C-PE presented higher *M*_w_ and *M*_n_ values than did the ^13^C–C-PE samples. These results indicate that copolymerization with hexene results in polymers with higher molar mass, likely due to the incorporation of larger hexene units. Furthermore, the homopolyethylene samples presented significantly lower *M*_n_ values than the copolymer samples, suggesting that the homopolyethylene has a higher proportion of lower molar mass polymer chains.

When the *Đ* values of the polyethylene materials were compared, homopolyethylene exhibited higher values (8.7 and 7.5) than copolyethylene (6.4 and 5.8). A higher *Đ* in homopolyethylene indicates a broader distribution of *M*_w_, which is expected due to its linear structure and more crystalline nature. The lower *Đ* values of the copolymers suggest a narrower molar mass distribution. Among the polyethylene materials, those produced with ^13^C monomers had lower *Đ* values compared to the corresponding PE materials produced with standard ethylene monomers alone.

DSC was used to determine the thermal properties *T*_m_, *T*_c_, and χ_c_ of the synthesized polyethylene samples (Table 2; the DSC thermograms are presented in Figures S2 and S3). Both homopolyethylene samples (^12^C–H-PE and ^13^C–H-PE) exhibit slightly higher melting temperatures compared to the copolyethylene samples (^12^C–C-PE and ^13^C–C-PE) due to their more crystalline structure. The incorporation of hexene comonomer in the copolymer introduces short side chains, disrupting the crystalline structure and leading to a lower melting temperature. Additionally, homopolyethylene shows higher crystallinity due to its regular, linear structure, while the copolymer has lower crystallinity and a more amorphous structure due to the presence of short side chains. Furthermore, homopolyethylene samples crystallize at temperatures slightly higher than those of copolymer materials. The presence of hexene in the copolymer lowers the crystallization temperature, indicating that the copolymer takes longer to organize into a crystalline structure upon cooling.

The TGA analysis results of polyethylene samples, such as onset of degradation, weight loss in TGA between 23 and 600 °C, and amount of inorganic residue at the end of TGA analysis, are also presented in Table 2. All PE samples exhibited a single decomposition peak, indicating one major thermal degradation event (Figures S4 and S5). This suggests that the material composition is relatively homogeneous and decomposes in a single step. Among the homopolyethylene materials, ^13^C–H-PE presented a slightly lower onset of decomposition, around 415 °C, while ^12^C–H-PE presented an onset of decomposition around 435 °C. It is clear from the values of the weight loss in TGA between 23 and 600 °C that the major composition of the polymer resins decomposes in this single step. The amount of inorganic residue in the samples after TGA at 1000 °C varied from 0.13 to 0.20 wt %.

The comonomer content in the ^12^C–C-PE sample was calculated from the ^13^C NMR analysis data.^44^ In the case of ^13^C–C-PE, the comonomer content was calculated by analyzing the methine peak, which corresponds to the branches originating from 1-hexene units, and the methylene peak from the polymer backbone. For the ^13^C–C-PE sample, the methine and methylene signals were normalized according to the ^13^C abundance of the respective (co)­monomer units, using the natural ^13^C abundance for 1-hexene-derived units and the calculated ^13^C abundance for ethylene-derived units. The comonomer content found in ^12^C–C-PE and ^13^C–C-PE was 0.3 and 0.2 mol %, respectively.

The synthesized polyethylene samples presented slight differences in FTIR spectra, especially in the fingerprint region (Figure 2). Here, absorption peaks at 1353, 1368, and 1378 cm^–1^ correspond to CH_2_ wagging vibrations for the amorphous polymer region, CH_2_ wagging vibrations for the crystalline region, and CH_3_ symmetrical bending vibrations, respectively.^45^

**Figure 2:**
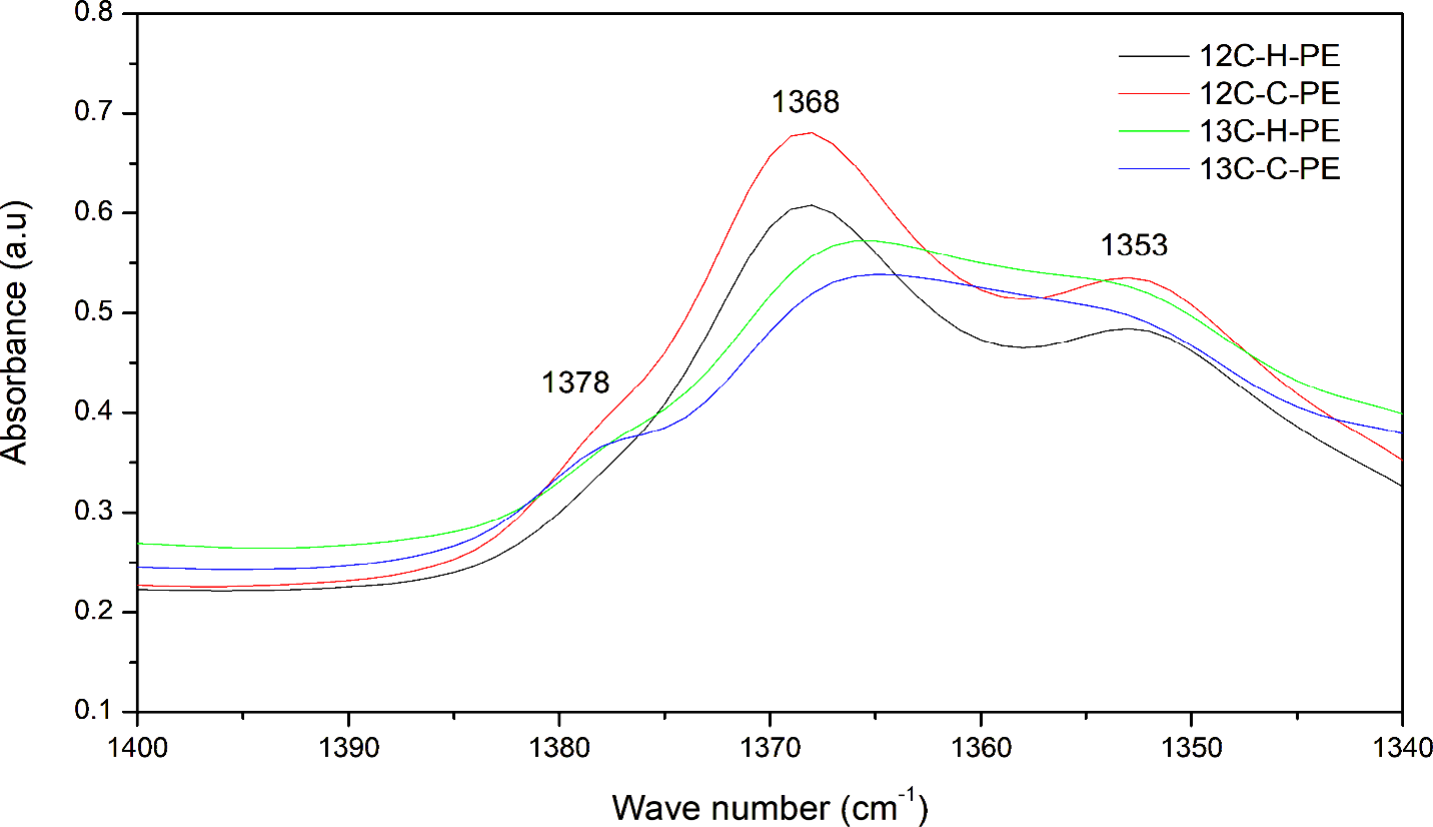
FTIR spectra of both ^13^C-enriched and conventional polyethylene samples in the frequency region 1400–1340 cm^–1^.

It is clear from the data that absorption peaks for CH_2_ wagging were prominent for homopolyethylene (^12^C–H-PE) and copolyethylene (^12^C–C-PE) produced with standard ethylene monomers, while ^13^C-enriched homopolyethylene (^13^C–H-PE) and copolyethylene (^13^C–C-PE) showed small shoulders. This observation could be due to the heavier CH_2_ in ^13^C-enriched polymers. The FTIR absorption peak caused by symmetric bending vibrations for CH_3_ at 1378 cm^–1^ is associated with the CH_3_ end groups in the short side chains in the polyethylene samples. The copolyethylene with ^13^C-enrichment (^13^C–C-PE) showed a clear shoulder at 1378 cm^–1^, while ^12^C–C-PE showed a weak shoulder, which could be due to interference of strong peaks ∼ 1365 to 1368 cm^–1^. The emergence of a CH_3_ shoulder confirms the findings from the DSC and NMR analyses regarding comonomer incorporation in both copolyethylene samples, as this peak is attributed to CH_3_ groups present in the side chains.

The FTIR spectra of the synthesized polyethylene samples were also examined in the 1550–1750 cm^–1^ frequency region (Figure 3). Both ^13^C-enriched and conventional homopolyethylene samples showed an FTIR absorption peak at 1635 cm^–1^ due to the C=C stretching vibration of the vinyl group, while the copolyethylene samples showed the absence of such a peak. The presence of such vinyl groups was not expected in these polyethylene samples as hydrogen was used during polymerization and may be due to incomplete hydrogenation.

**Figure 3:**
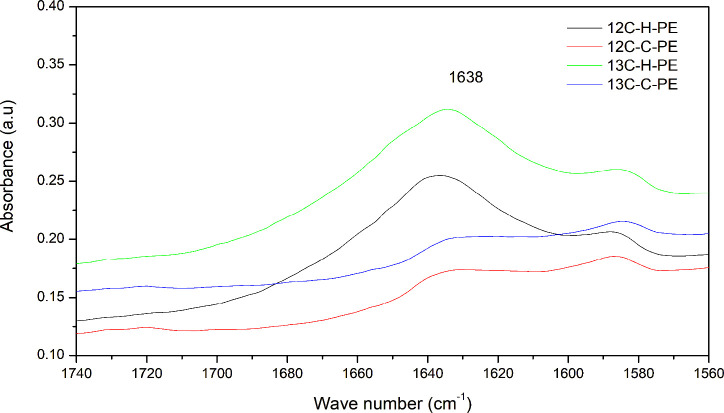
FTIR spectra of both ^13^C-enriched and conventional polyethylene samples in the frequency region 1740–1560 cm^–1^.

The FTIR spectra of the synthesized polyethylene samples were further examined between the frequency regions of 1850 and 1950 cm^–1^ (Figure 4). Both ^13^C-enriched homopolyethylene and copolyethylene samples showed an FTIR absorption peak at around 1890 cm^–1^, which was shifted to a higher frequency region compared to conventional homopolyethylene and copolyethylene samples that show a peak at 1897 cm^–1^. The presence of such an absorption peak in PE has also been reported by others.^45^

**Figure 4:**
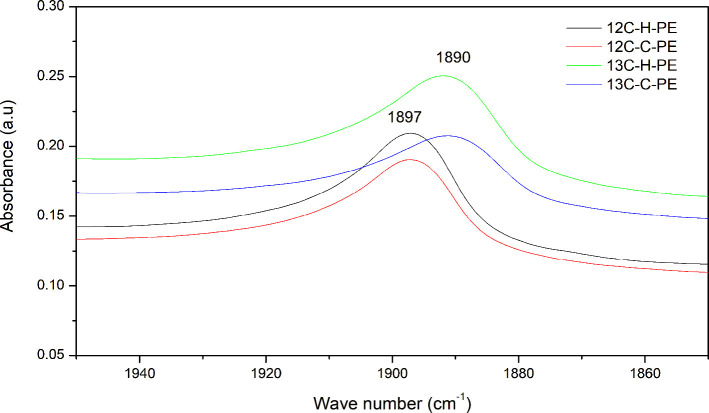
FTIR spectra of both ^13^C-enriched and conventional polyethylene samples in the frequency region 1950–1850 cm^–1^.

### 3.2 Determination of ^13^C-Enrichment in PE Materials

The ^13^C-enrichment in the polyethylene samples was determined by using nuclear magnetic resonance (NMR) and IRMS methods. NMR can be used to determine ^13^C-enrichment in PE samples by measuring the total magnetization intensity of the ^13^C spins in the polyethylene backbone per mass of the sample. The ^13^C NMR spectra of the reference polyethylene and ^13^C-enriched polyethylene samples are presented in Figure S6. By knowing the magnetization intensity-to-mass ratio for the ^13^C-enriched samples and comparing it to reference samples with natural ^13^C abundance, it is possible to calculate the ^13^C content in the samples.^46^ The calculated ^13^C contents in ^13^C–H-PE and ^13^C–C-PE were 25 and 23 mol %, respectively (Table 3). These measurements were in good agreement with the expected values as we anticipated a value close to 25%. To further confirm the ^13^C mol %, we estimated the ^13^C content using IRMS as a second method.

**Table 3:** ^13^C NMR and IRMS Analysis Results for ^13^C–H-PE and ^13^C–C-PE

sample	^13^C NMR results	IRMS analysis results			
	^13^C (mol %)	δ^13^C (‰) (SD)	^13^C/^12^C ratio (SD/RSD)	^13^C (mol %)	*n*
^13^C–H-PE	25	21,889 (65)	0.256 (0.10/0.39)	20.26	3
^13^C–C-PE	23	24,607 (580)	0.286 (0.64/2.25)	22.26	3

The IRMS analysis provides precise quantification of the ^13^C/^12^C isotope ratios in materials. IRMS analysis was conducted on reference unlabeled polyethylene materials and ^13^C-enriched polyethylene materials. The reference samples, ^12^C–H-PE and ^12^C–C-PE, exhibited δ^13^C values of −36.2 ± 0.2 and −38.9 ± 0.2‰, respectively, confirming the absence of ^13^C-enrichment. The IRMS results for ^13^C-enriched polyethylene samples, including δ^13^C values, ^13^C/^12^C ratios, and ^13^C mol % for both materials, are summarized in Table 3. The ^13^C/^12^C ratio and ^13^C mol % in the ^13^C-enriched samples were calculated according to the established methods.^47,48^ In the ^13^C–H-PE sample, the carbon content and ^13^C/^12^C ratio indicate significant ^13^C-enrichment, with low standard deviation (SD) and relative standard deviation (RSD) values. The calculated amount of ^13^C-enrichment in this sample was 20.26 mol %. Similarly, the ^13^C–C-PE sample exhibited lower carbon content but a higher degree of ^13^C-enrichment compared to ^13^C–H-PE. The measurements for ^13^C/^12^C ratios in ^13^C–C-PE show a bit more variability than those in ^13^C–H-PE, yet remain within an acceptable range for isotopic analysis, indicating reliable measurements. The ^13^C-enrichment in this sample was determined to be 22.26 mol %.

### 3.3 Py-GCMS Analysis of the ^13^C-Enriched PE Samples

The pyrolysis of the PE materials at 600 °C generated a range of typical pyrolysis products, reflecting the polymer structure (Figure 5). Py-GCMS analysis of the polymer samples produced peaks corresponding to oligomeric fragments from C6 to C36, consistent with previously reported studies.^49^ The pyrolysis fingerprint confirmed the presence of triplet patterns representing diolefins, olefins, and *n*-paraffins, with chain lengths ranging from C9 to C24. The triplet peaks were clearly identified within this range, providing evidence of a well-defined fragmentation behavior. However, for longer-chain homologues (above C24), peak resolution was limited due to coelution, making it difficult to distinguish individual triplet patterns in this region.^49,50^

**Figure 5:**
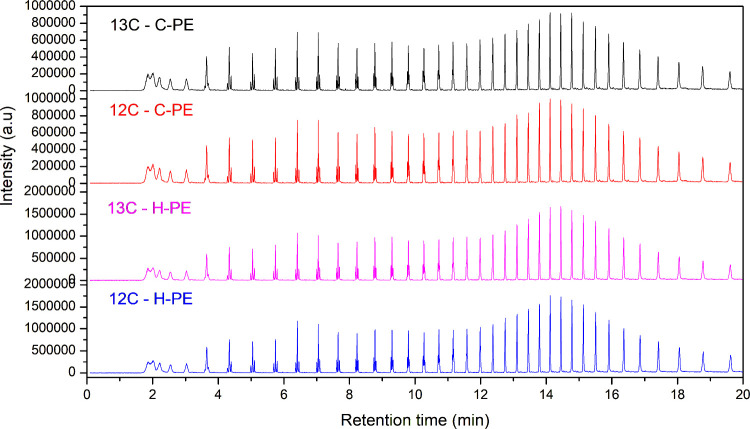
TIC pyrograms of ^12^C–H-PE, ^13^C–H-PE, ^12^C–C-PE, and ^13^C–C-PE samples between the retention times of 0 and 20 min.

The pyrograms of both ^12^C- and ^13^C-labeled PE samples (Figure 6) appear similar, confirming that the pyrolysis behavior and product distribution are consistent across both samples. However, the mass spectra of individual compounds show significant differences due to the incorporation of ^13^C in the labeled sample. For example, dodecane (C12, molar mass: 170) is one of many peaks observed, with pyrolysis fragments ranging up to C46. The mass spectra for ^12^C-labeled dodecane showed predominant fragment ions at *m*/*z* 29, 41, 43, 57, 71, and 85 (Figure 7). In contrast, the ^13^C-labeled dodecane exhibited a broader set of fragment peaks at *m*/*z* 29, 41, 42, 43, 44, 55, 56, 57, 58, 70, 71, 72, 84, 85, 86, 98, and 99, indicating the presence of ^13^C in various positions within the molecule (Figure 8). When zooming in on the molecular ion region, we observed that the *m*/*z* 170 and 170.4 peaks were dominant for ^12^C-dodecane (Figure 7), while the ^13^C-labeled dodecane sample displayed a distinct peak at *m*/*z* 171.9, with no *m*/*z* 170 peak present (Figure 8). This absence of the *m*/*z* 170 peak and the shift to higher masses in the ^13^C sample provide clear evidence of the incorporation of ^13^C in the polymer structure.

**Figure 6:**
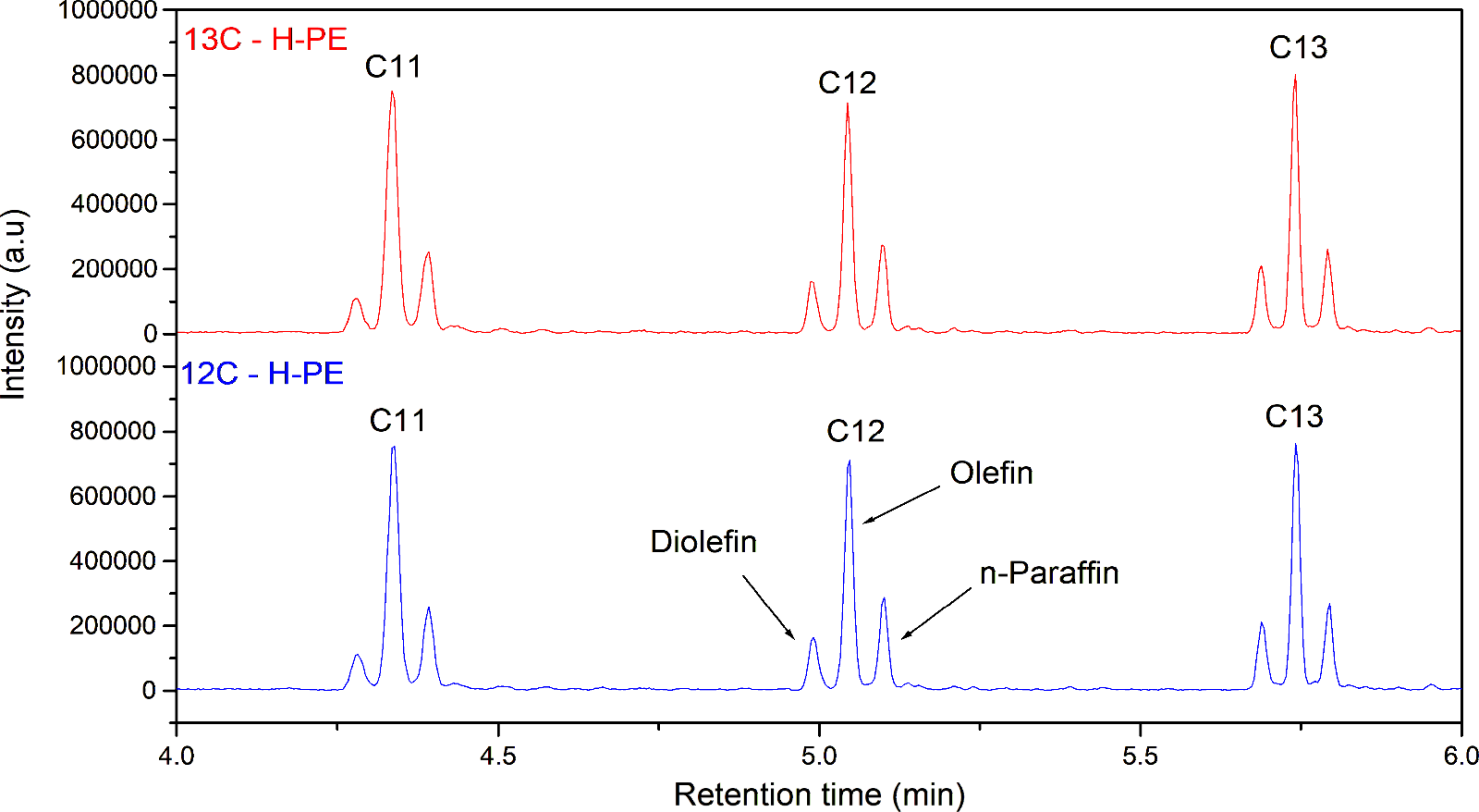
TIC pyrograms of ^12^C–H-PE and ^13^C–H-PE samples between the retention times of 4 and 6 min.

**Figure 7:**
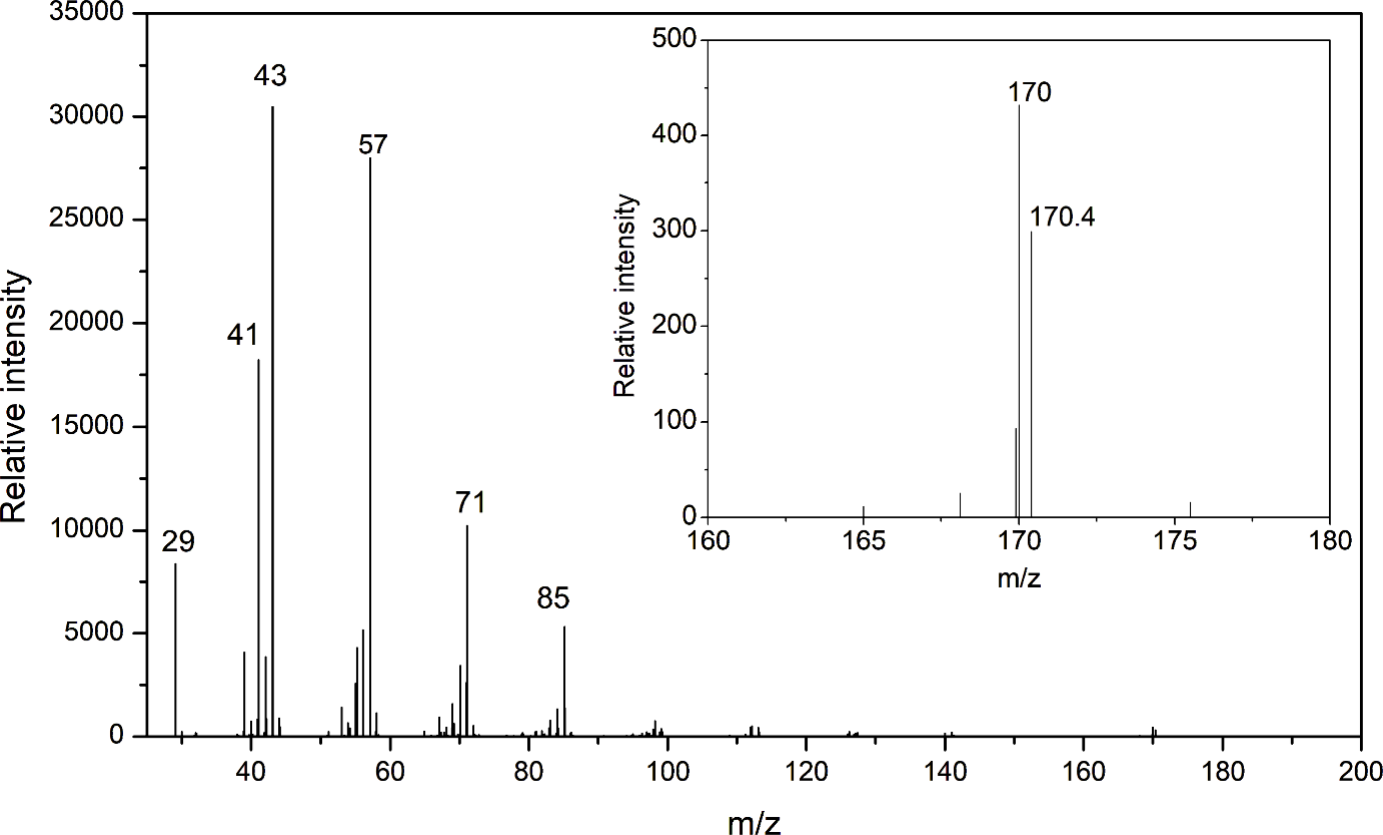
Mass spectrum of the dodecane fragment from ^12^C–H-PE. Inset image shows the mass spectrum of molecular ions of dodecane in the same sample.

**Figure 8:**
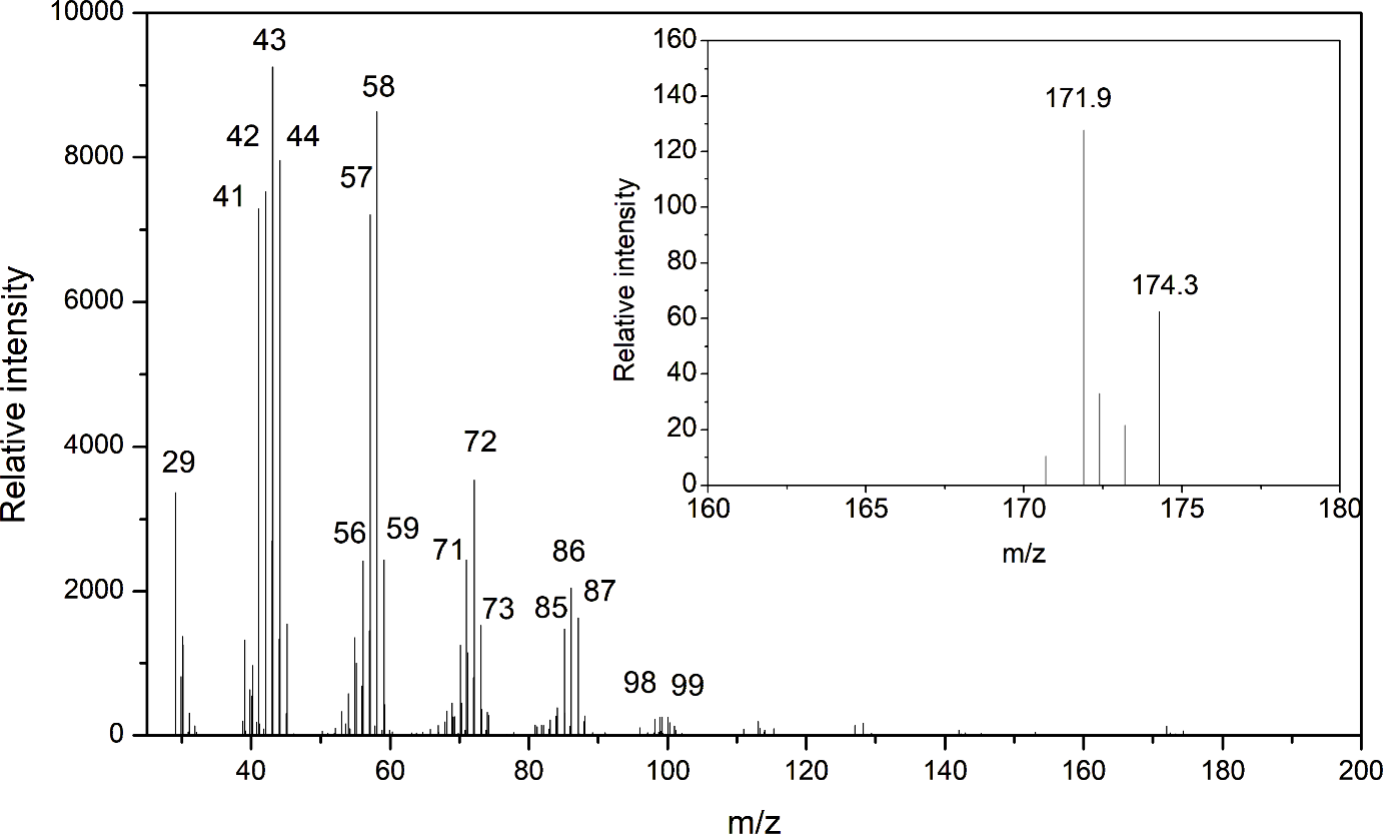
Mass spectrum of the dodecane fragment from ^13^C–H-PE. Inset image shows the mass spectrum of the molecular ion of dodecane in the same sample.

The quantification of total ^13^C/^12^C ratios in neat polyethylene (PE) samples using Py-GCMS is not straightforward due to several analytical and instrumental limitations. Unlike IRMS, which is specifically designed for high-precision isotope ratio measurements, standard GCMS instruments and workflows are not optimized for isotope quantification at the whole-molecule or total carbon level. The two key challenges associated with direct isotope quantification in PE when using Py-GCMS are as follows. (1) Extensive fragmentation during electron ionization (EI): In typical Py-GCMS, EI is used, which often results in extensive fragmentation of the pyrolysis products. As a result, the molecular ion (M^+^) peak is often weak or even absent, making it difficult to track intact isotopologues of specific chain lengths.^37,51^ (2) A complex mixture of pyrolysis products: Pyrolysis of PE generates a wide range of oligomeric fragments, including alkanes, alkenes, and alkadienes, typically ranging from C6 to C48. These compounds coelute and overlap in the chromatogram, creating complex spectra where isotopic patterns are often convoluted.^38^ The complexity increases the risk of peak interference and isotope ratio distortion.

To overcome this challenge of using Py-GCMS, the oxidation of PE samples was performed to enable the determination of ^13^C/^12^C isotope ratios and to investigate the formation of oxidation products. The oxidation process introduced oxygen-containing functional groups such as ketones, aldehydes, and carboxyl groups.^52^ The formation of oxygen-containing components in oxidized PE samples was confirmed by analyzing the pyrograms in the extracted ion chromatogram (EIC) mode at *m*/*z* 58, 59, and 60. The *m*/*z* 58 signal serves as a diagnostic marker for methyl ketones in the oxidized PE.^53^ The total ion chromatogram (TIC) and EICs of oxidized ^12^C–H-PE and ^13^C–H-PE are shown in Figure 9 and Figure 10, respectively. Both oxidized PE samples exhibited prominent peaks corresponding to *m*/*z* 58. Additionally, the elution of *m*/*z* 59 supports the presence of methyl ketone functional groups.^53^ In the case of ^13^C–H-PE, an additional signal at *m*/*z* 60 was observed, corresponding to ^13^C-labeled analogues of methyl ketones. The coelution of *m*/*z* 58 and 59, along with the exclusive appearance of *m*/*z* 60 in the ^13^C–H-PE sample, confirms the identification of these peaks as methyl ketones and further validates the incorporation of ^13^C.

**Figure 9:**
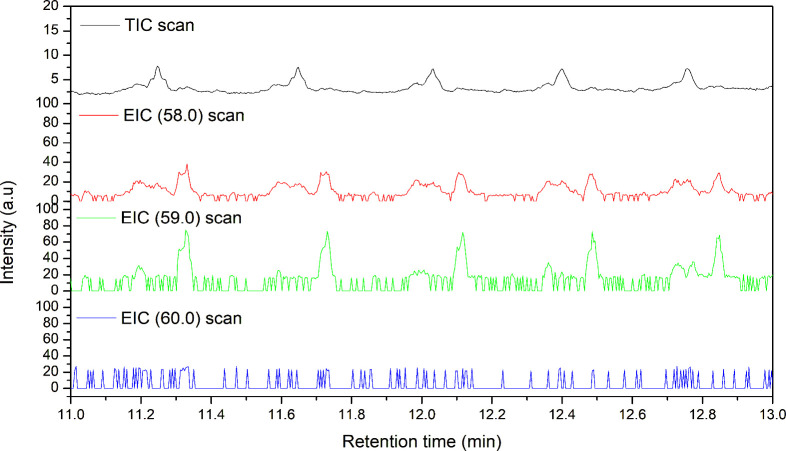
TIC and EIC of *m*/*z* 58, 59, and 60 of the oxidized ^12^C–H-PE sample from a retention time of 11–13 min.

**Figure 10:**
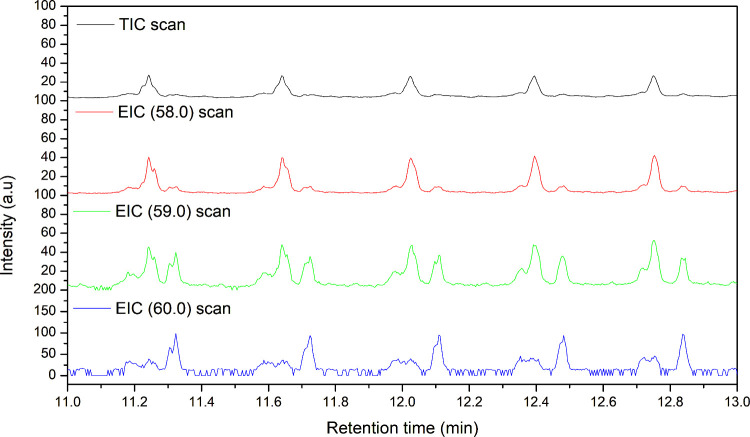
TIC and EIC of *m*/*z* 58, 59, and 60 of the oxidized ^13^C–H-PE sample from a retention time of 11–13 min.

The pyrograms of virgin and oxidized ^13^C–H-PE are presented in Figure 11. Clear differences were observed between the two, particularly at lower retention times. In the oxidized ^13^C–H-PE sample, a dominant CO_2_ peak eluting early in the chromatogram indicates the formation of CO_2_ as a result of the thermal degradation of oxygenated species introduced during the oxidation step. This CO_2_ formation enables quantification of ^13^C-enrichment by measuring the peak intensities at *m*/*z* 44 (^12^CO_2_) and *m*/*z* 45 (^13^CO_2_).

**Figure 11:**
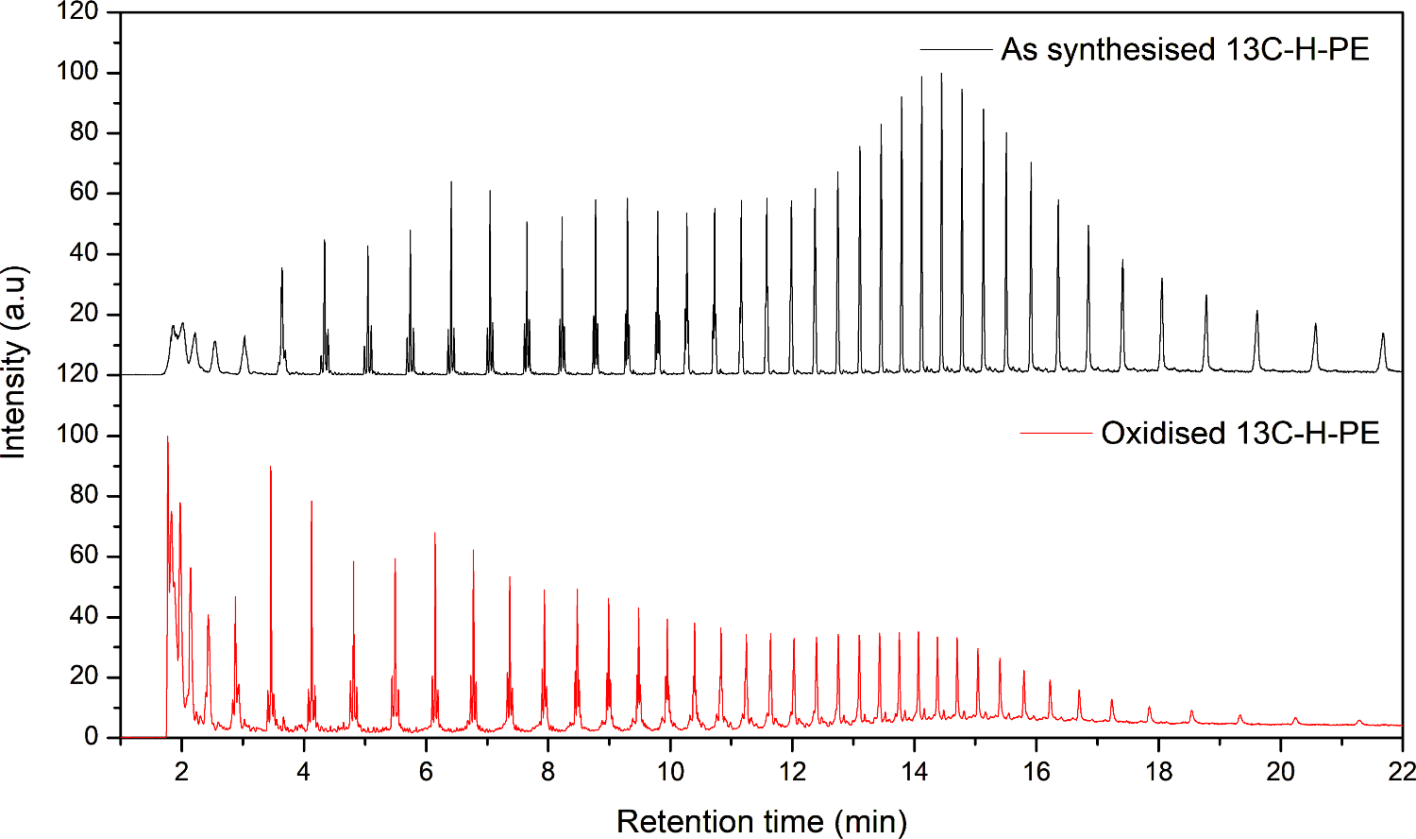
TICs of the as-synthesized and oxidized ^13^C–H-PE samples.

### 3.4 Determination of ^13^C-Enrichment by Py-GCMS

The quantification of ^13^C-enrichment in PE samples was achieved by measuring the area of the *m*/*z* 45 (^13^CO_2_) peak in Py-GCMS. Although Py-GCMS is typically conducted under an inert carrier gas atmosphere such as helium (He) to prevent combustion, CO_2_ can still be formed and detected when analyzing preoxidized PE samples. The oxygen-containing functional groups such as carboxylic acids, aldehydes, ketones, and alcohols introduced during preoxidation of PE are responsible for the formation of CO_2_. At the pyrolysis temperature, 600 °C, these oxygen-containing groups undergo thermal degradation that results in fragmentation and decarboxylation, releasing CO_2_ molecules without the need for molecular oxygen (O_2_) in the pyrolysis atmosphere. For example, the carboxylic acids and esters readily decarboxylate upon thermal decomposition to form a CO_2_ and hydrocarbon fragments. Similarly, the aldehydes and ketones can also rearrange or oxidize further, especially under high-temperature pyrolytic conditions, resulting in CO and CO_2_ formation. This mechanism is well-documented in the polymer degradation literature, where oxidized or weathered polyethylene is shown to yield small molecules including CO, CO_2_, and H_2_O as part of the breakdown process.^33,52^ During the Py-GCMS analysis, these volatile products are carried by helium into the GC column and detected by the MS. The CO_2_ appears as a sharp early eluting peak, and its isotopologues can be identified via their characteristic mass-to-charge ratios, *m*/*z* 44 for ^12^CO_2_ and *m*/*z* 45 for ^13^CO_2_. Thus, even in the absence of atmospheric oxygen, the in situ decomposition of preoxidized functionalities in the polymer leads to the formation of detectable CO_2_, enabling the quantitative evaluation of ^13^C/^12^C ratios using Py-GCMS.

To ensure reliable and reproducible ^13^C/^12^C isotope ratio determination, the oxidation process was optimized by testing different oxidation times for both ^12^C and ^13^C homopolyethylene samples at 200 °C. The oxidation time was systematically increased, and the resulting CO_2_ intensities were analyzed to determine the point at which isotope ratios stabilized. The goal was to determine the minimum oxidation time required to achieve stable CO_2_ formation and consistent isotope ratio measurements. This optimization was crucial to achieving reproducible results. The reference PE sample, ^12^C–H-PE, was oxidized for 20, 40, and 60 min and analyzed using Py-GCMS, and the resulting CO_2_ signals were monitored (Table 4). The results showed that the ^13^C/^12^C isotope ratio remained constant at 1.2% across all oxidation durations, indicating that further oxidation beyond 20 min did not introduce additional ^13^C-enrichment or degradation. This confirmed that the reference sample maintained its expected natural isotopic composition, validating the oxidation process.

**Table 4:** ^13^C/^12^C Ratio in Thermally Oxidized ^12^C–H-PE and ^13^C–H-PE for Different Durations

sample	oxidation time (min)	^12^CO_2_ (44) base peak intensity (%)	^13^CO_2_ (45) base peak intensity (%)	^13^C/^12^C ratio (%)	^13^C (mol %)
^12^C–H-PE	20	100	1.2	1.2	1.18
^12^C–H-PE	40	100	1.2	1.2	1.18
^12^C–H-PE	60	100	1.2	1.2	1.18
^13^C–H-PE	20	100	14.3	14.3	12.51
^13^C–H-PE	40	100	24.1	24.1	19.41
^13^C–H-PE	60	100	25.4	25.4	20.25
^13^C–H-PE	80	100	25.5	25.5	20.31
^13^C–H-PE	100	100	25.1	25.1	20.06

Similarly, the optimal oxidation times for the ^13^C-labeled PE and ^13^C–H-PE samples were 20, 40, 60, 80, and 100 min, and the intensities of ^12^CO_2_ (*m*/*z* 44) and ^13^CO_2_ (*m*/*z* 45) were measured (Table 4). The ^13^C/^12^C isotope ratio gradually increased from 14.3% at 20 min to 25.4% at 60 min. With a further increase in oxidation time, longer than 60 min, minor variations in the ^13^C/^12^C ratio suggested that the system had reached equilibrium. Based on these findings, 70 min at 200 °C was selected as the standard oxidation time, ensuring complete oxidation and stable isotope ratio measurements.

To quantify the ^13^C/^12^C isotope ratios in PE materials, oxidized polymer samples were analyzed by using Py-GCMS. The intensity of ^13^CO_2_ (*m*/*z* 45) and ^12^CO_2_ (*m*/*z* 44) peaks was used to determine the isotope ratios in different polymer samples (Figure 12). The results are summarized in Table 5. The reference PE sample ^12^C–H-PE was analyzed to confirm natural ^13^C abundance levels, which remained consistent at 1.18 mol % across both replicates. In contrast, the ^13^C-labeled PE sample exhibited significantly higher isotope ratios, averaging 25.4%. The precision of this method was evaluated by conducting six replicate measurements, which resulted in an RSD of 1.1%, demonstrating excellent repeatability. Both copolymer samples ^12^C–C-PE and ^13^C–C-PE were also analyzed after oxidation. The reference copolymer ^12^C–C-PE exhibited a natural ^13^C isotope abundance of 1.18 mol %. The ^13^C-labeled copolymer ^13^C–C-PE had an average isotope ratio of 28.4%, with an RSD of 0.7%. These results further confirmed the robustness of Py-GCMS for precise isotope ratio determination in different polymer compositions. The ^13^C isotope enrichment values in ^13^C–H-PE and ^13^C–C-PE samples were 20.22 and 22.13 mol %, respectively.

**Figure 12:**
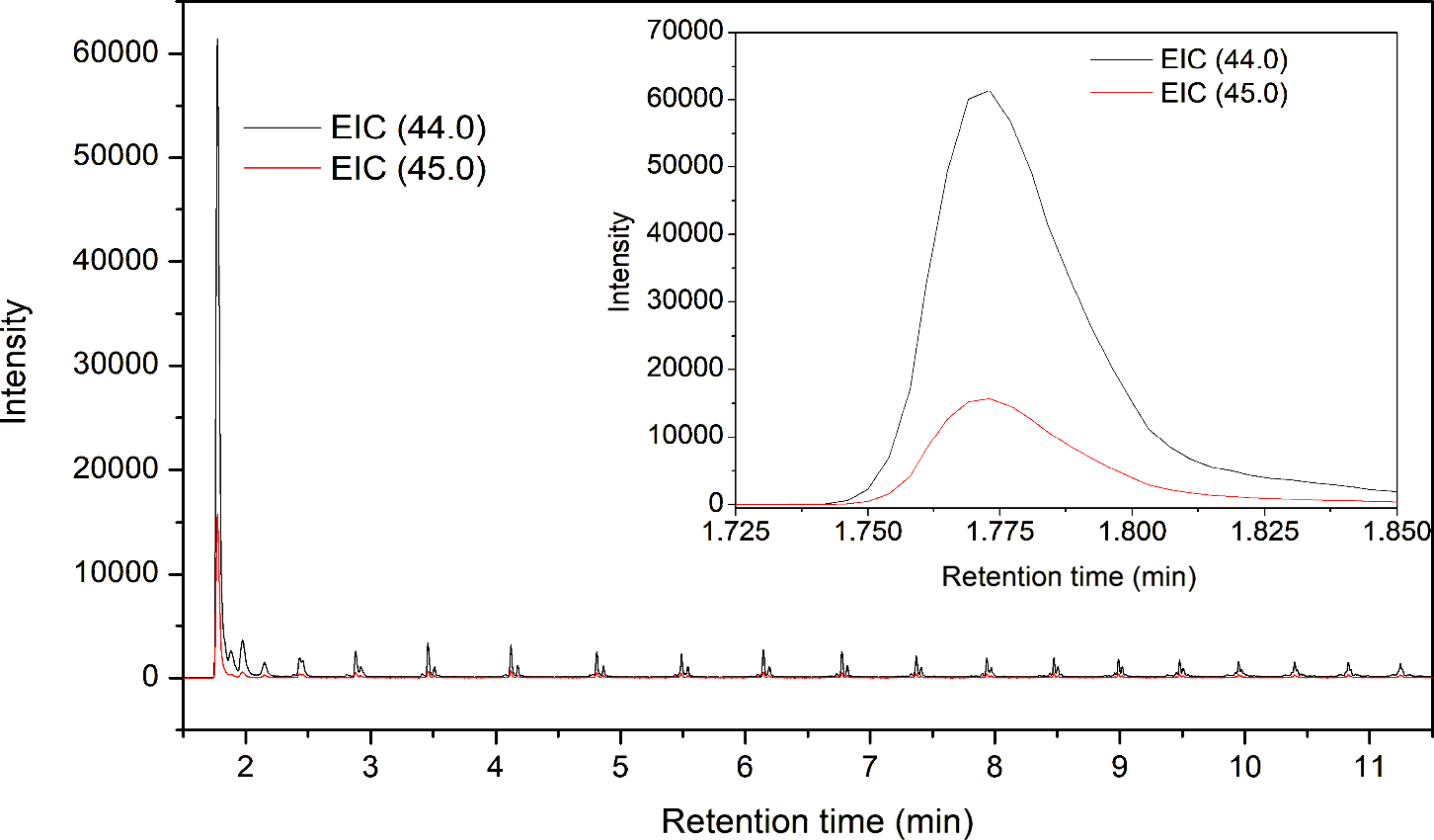
EIC of CO_2_ (*m*/*z* 44 and 45) from thermally oxidized ^13^C–H-PE. Inset image shows the close-up of EIC of CO_2_.

**Table 5:** Average ^13^C/^12^C Ratio in Thermally Oxidized PE Materials by Py-GCMS Analysis

sample	^12^CO_2_ (*m*/*z* 44) intensity	^13^CO_2_ (*m*/*z* 45) intensity	^13^C/^12^C ratio (SD/RSD)	^13^C (mol %)	*n*
^12^C–H-PE	100	1.2	1.20 (0.00)	1.18	2
^13^C–H-PE	100	25.35	25.35 (0.28/1.1)	20.22	6
^12^C–C-PE	100	1.2	1.25 (0.07)	1.18	2
^13^C–C-PE	100	28.42	28.42 (0.19/0.7)	22.13	6

The data show a clear distinction in isotope incorporation between the ^13^C–H-PE and ^13^C–C-PE. The ^13^C-labeled copolymer exhibited a slightly higher ^13^C/^12^C ratio (28.4%) compared to the ^13^C-labeled homopolymer (25.4%), suggesting potential variations in the incorporation efficiency of ^13^C monomers during polymerization. These findings highlight the capability of Py-GCMS to distinguish isotopic compositions in different polymer structures. The reproducibility of the results, indicated by low SD and RSD values, confirms that Py-GCMS is a robust method for ^13^C/^12^C ratio determination in polymers. Additionally, the determination of ^13^C-enrichment in ^13^C-enriched polyethylene samples using Py-GCMS analysis shows a strong correlation with values obtained through the IRMS method, suggesting that Py-GCMS serves as a credible alternative to conventional IRMS techniques.

Future research will investigate the degradation mechanisms of polyethylene in various environments by monitoring ^13^C in the synthesized ^13^C-enriched polyethylene samples.

## 4 Conclusions

Polyethylene samples with varying microstructures and ^13^C-enrichment were successfully synthesized by polymerizing ethylene monomers naturally abundant or enriched in the ^13^C isotope. The PE samples were characterized with respect to molar mass, melting and crystallization temperatures, crystallinity, thermal stability, chemical composition, and ^13^C-enrichment. Polyethylene samples synthesized with approximately 25% ^13^C-enriched ethylene monomers exhibited microstructural characteristics similar to those of conventional polyethylene. Although slight variations in molar mass were observed due to differences in monomer incorporation, all samples had comparable thermal stability.

The lower crystallization temperatures and reduced crystallinity observed for copolyethylene materials in the DSC analysis, along with the appearance of an absorption peak attributed to CH_3_ end groups at 1378 cm^–1^ in the FTIR spectra, indicated incorporation of a comonomer into the polyethylene. FTIR analysis further elucidated the chemical compositional variations in the microstructure of the produced polyethylene samples.

Quantification of ^13^C-enrichment via IRMS yielded enrichment levels of approximately 25.6% in homopolyethylene (^13^C–H-PE) and 28.6% in copolyethylene (^13^C–C-PE). In contrast, ^13^C NMR analysis provided similar enrichment in homopolyethylene but indicated around 5% lower enrichment in the copolyethylene sample. The Py-GCMS analysis of partially oxidized polyethylene yielded ^13^C-enrichment values comparable to those obtained by IRMS, supporting the use of Py-GCMS for ^13^C-enrichment quantification.

Taken together, our data demonstrated that the properties of ^13^C-enriched polyethylene are virtually indistinguishable from those of polyethylene synthesized from conventional ethylene, making it a suitable material for soil microplastic mineralization experiments without the risk of artifacts arising from physicochemical deviations from standard PE.

## Supplementary Material



## Data Availability

Data will be made available on request.

## Notes

Figure S1: molar mass distribution curves for polyethylene samples; Figure S2: second heating DSC thermograms of polyethylene samples; Figure S3: cooling DSC thermograms of polyethylene samples; Figure S4: TGA weight loss patterns for polyethylene samples; Figure S5: TGA derivative weight loss patterns for polyethylene samples; and Figure S6: ^13^C NMR spectra of polyethylene samples (PDF)

## §

Amiblu Technology AS, Østre Kullero̷d 3, NO-3241 Sandefjord, Norway
