# Supplementary material for: Laboratory Synthesis, Characterization, and Py-GCMS-Based 13C Quantification of 13C‑Enriched Polyethylene
Source: ACS Omega. 2026 May 5;11(19):28368–80. doi: 10.1021/acsomega.6c00354 (PMC13191553; doi:10.1021/acsomega.6c00354)
Supplement: Supplementary file 1 [file ao6c00354_si_001.pdf]

Laboratory Synthesis, Characterization, and Py-GCMS-based ^{13}C Quantification of ^{13}C-Enriched Polyethylene

*Ravindra Reddy Chowreddy[1], Alireza Hassani[†1], Gustav Vaaje-Kolstad[*2] and Bavan Mylvaganam[1]*

[1]Norner Research AS, Dokkvegen 20, 3920 Porsgrunn, Norway

[2]Norwegian University of Life Sciences, Faculty of Chemistry, Biotechnology and Food Science, 1433 Ås, Norway

*Corresponding author

E-mail: gustav.vaaje-kolstad@nmbu.no

[†]Present address: Amiblu Technology AS, Østre Kullerød 3, NO-3241 Sandefjord, Norway

Supporting information

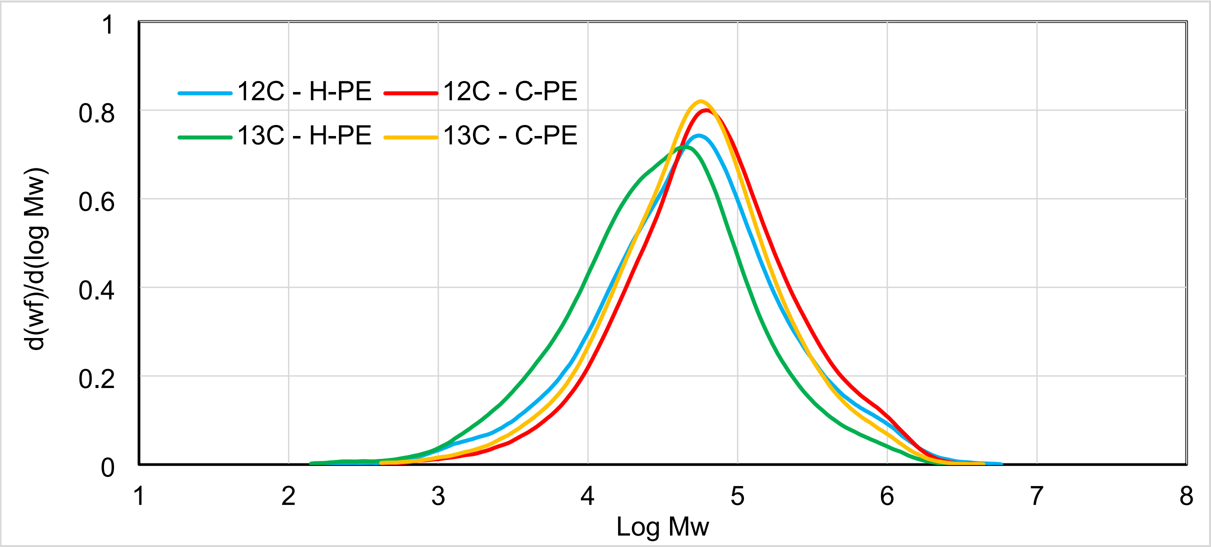

Figure S 1. Molar mass distribution curves for polyethylene samples

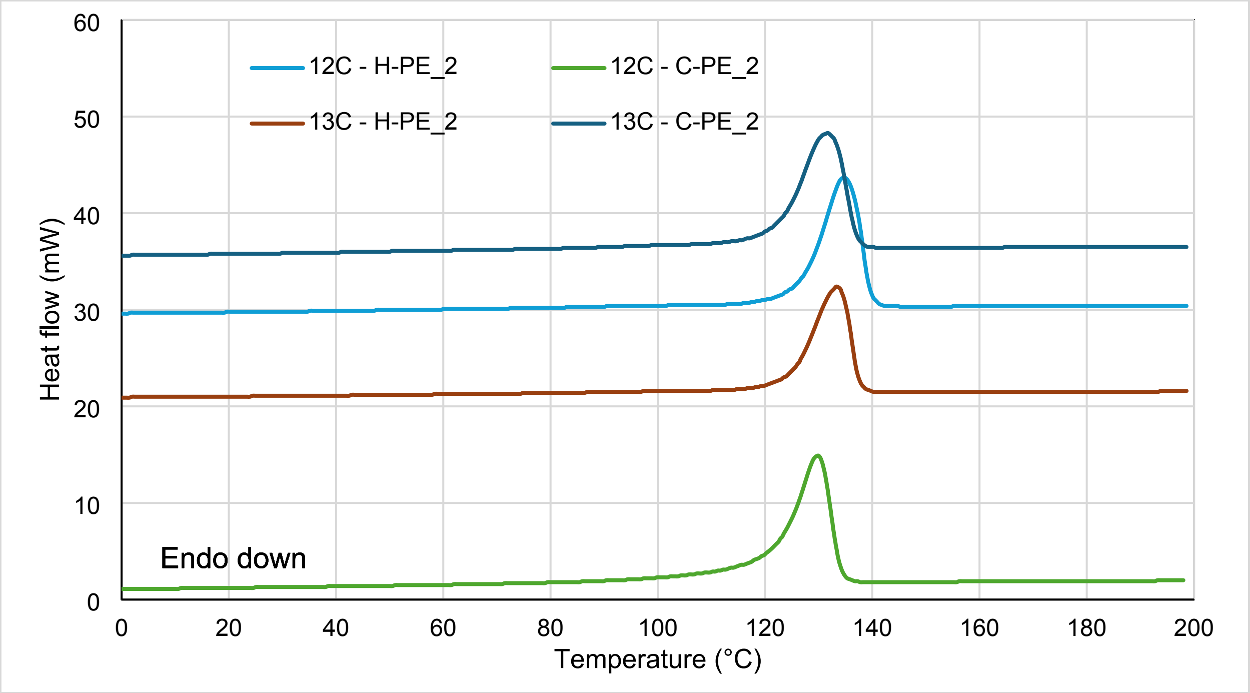

Figure S 2. Second heating DSC thermograms of polyethylene samples

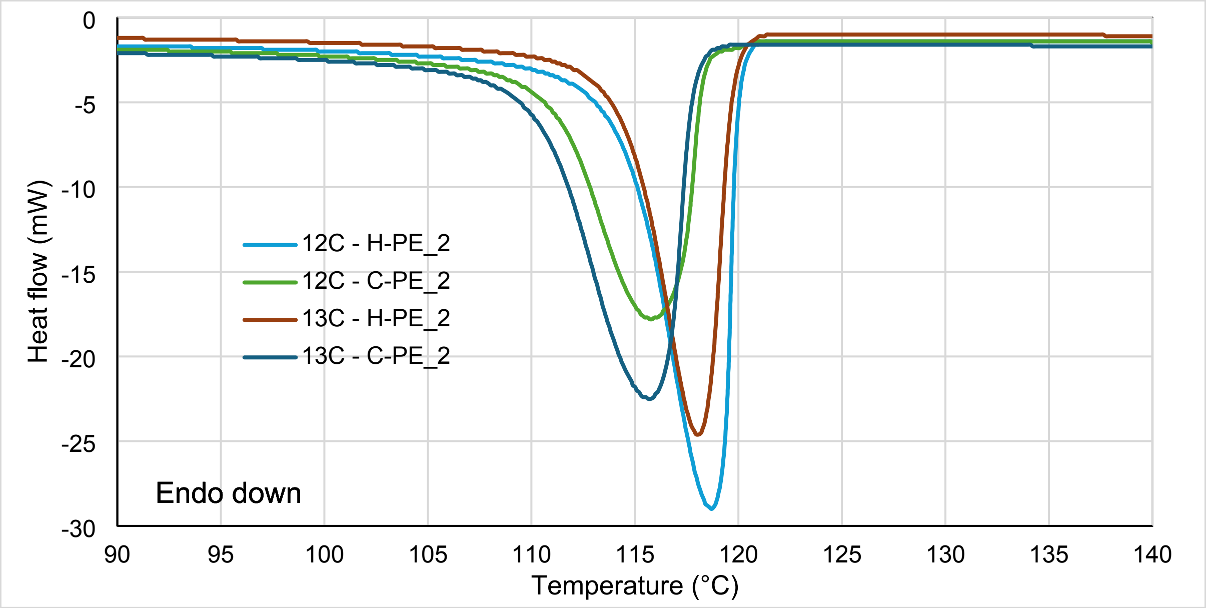

Figure S 3. Cooling DSC thermograms of polyethylene samples

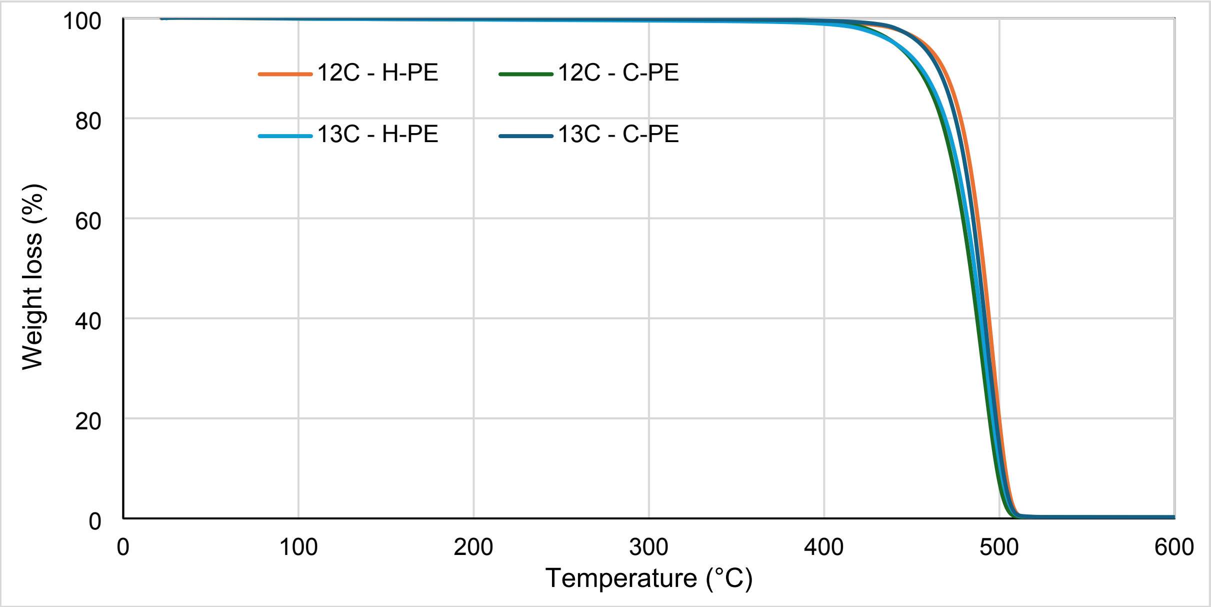

Figure S 4. TGA weight loss patterns for polyethylene samples

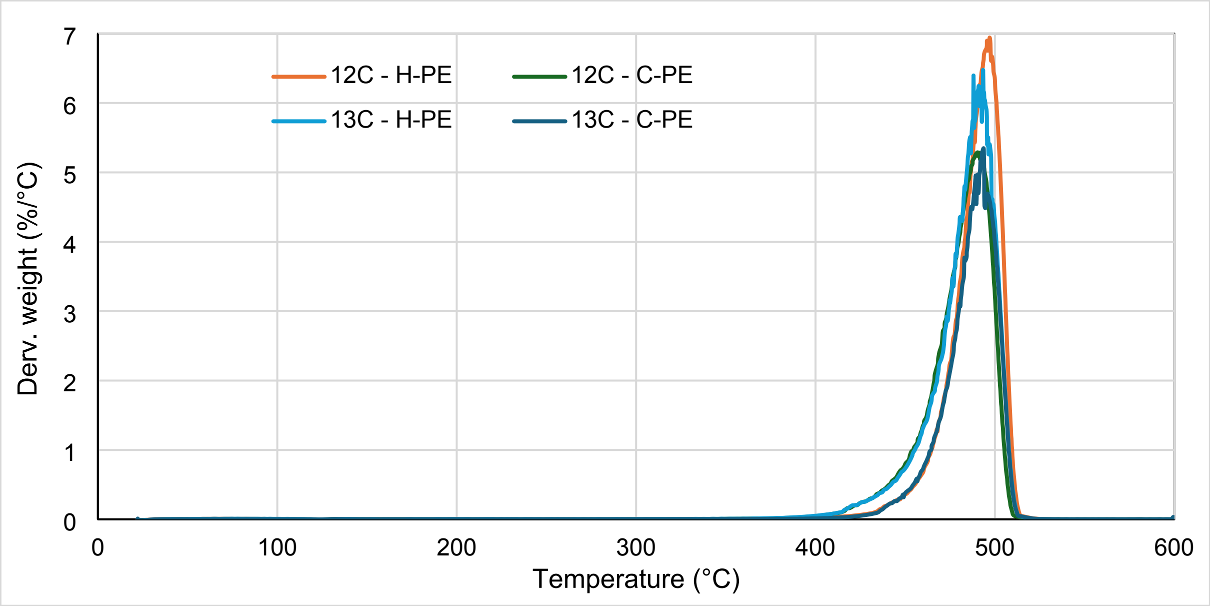

Figure S 5. TGA derivative weight loss patterns for polyethylene samples

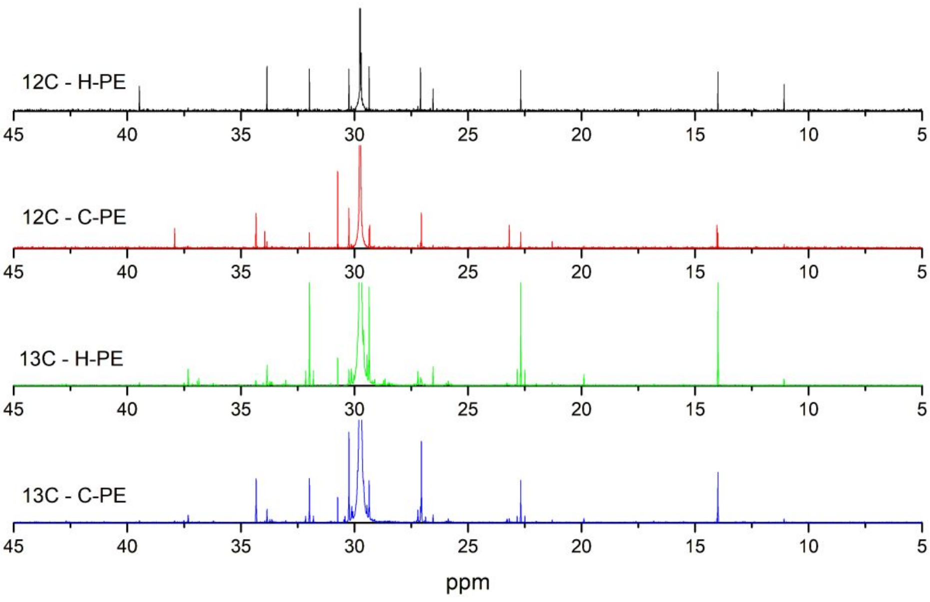

Figure S 6. ^{13}C NMR spectra of polyethylene samples
